# Support perceived by foreign-origin women in Finnish maternity care: a comparison of two surveys

**DOI:** 10.1093/eurpub/ckac131.436

**Published:** 2022-10-25

**Authors:** A Schmidt, A Chen, R Klemetti

**Affiliations:** 1 Finnish Institute for Health and Welfare, Helsinki, Finland; 2 Aalto University, Espoo, Finland; 3 Rutgers, Robert Wood Johnson Medical School, Piscataway, USA

## Abstract

**Background:**

Evidence suggests that foreign-origin women are at risk of poorer pregnancy outcomes, both worldwide and in Finland. This study examined if foreign-origin women felt adequately supported by healthcare providers during their perinatal care in Finland.

**Methods:**

Foreign-origin women who gave birth in Finland within the last 3 years were recruited via snowball method as part of the MOM Survey (MS). Data were also extracted from birthing parents’ responses to the 2020 national FinChildren Survey (FC), which consisted of data from parents in Finland with 3-6-month-old babies. Only responses submitted to MS from women born outside of Finland (n = 291) and responses submitted to FC by women born in Finland (n = 7984) were analyzed. Reported levels of adequate support from each group were compared using an independent sample proportion t-test. Data were then pooled and analyzed via binomial logistic regression.

**Results:**

MS respondents were older, more highly educated, and reported a higher proportion of single parenthood. 70.9% of MS respondents reported receiving adequate support regarding general well-being during pregnancy, compared to 90.2% of FC respondents (p < 0.001). Statistically significant differences (p < 0.001) were also seen in levels of support for parenthood (MS 74.3%, FC 92.8%), depression (MS 68.1%, FC 94.7%), fear of childbirth (MS 81.5%, FC 91.7%), and preparing for childbirth (MS 65.0%, FC 80.6%). After adjusting for age, education, and relationship status, FC mothers were still 4 to 9 times more likely to report receiving adequate support in these areas as compared to their MS counterparts.

**Conclusions:**

Women born outside of Finland were significantly more likely to report receiving inadequate support in multiple aspects of their perinatal care when compared to Finnish-born women. This held true even after adjusting for demographic differences. More research is needed to better understand this phenomenon and to ensure equitable care in the future.

**Key messages:**

• Minority populations continue to grow in Finland, a country often seen as a global leader in maternal care. Supporting these populations is imperative to ensure quality maternal healthcare for all.

• Our observation that foreign-origin women felt less supported during pregnancy suggests that new care models or strategies may be needed to address their unique needs in peripartum care.

